# Weak evidence of trade-offs modulated by seed mass among a guild of closely related winter annuals

**DOI:** 10.1007/s00442-023-05416-8

**Published:** 2023-07-12

**Authors:** Isis A. da Silva, Margaret M. Mayfield, John M. Dwyer

**Affiliations:** 1 School of Biological Sciences, The University of Queensland, St Lucia, QLD Australia; 2 School of Biological Sciences, The University of Melbourne, Parkville, VIC Australia

**Keywords:** Seed mass, Plant interactions, Survival, Biomass, Mediterranean

## Abstract

Plant-plant interactions are integral to the establishment and persistence of diversity in plant communities. For annual plant species that depend on seeds to regenerate, seed characteristics that confer fitness advantages may mediate processes such as plant-plant interactions. Seed mass is known to vary widely and has been shown to associate with species’ differences in stress tolerance and competitive effects. However, understanding of how seed mass influences species’ responses to competition is less well understood. Using natural assemblages of six closely related annual plant species in Western Australia, we implemented a thinning study to assess how seed mass influences the outcomes of plant-plant interactions. We found relatively weak evidence for competition or facilitation among species. Our strongest results indicated that heavy-seeded species had lower survivorship than light-seeded species when interacting with heterospecifics. Seed mass was also negatively related to overall survival, counter to expectations. These findings indicate some evidence for trade-offs mediated by seed mass in this system. However, we acknowledge that other factors may have influenced our results, such as the use of natural assemblages (rather than using sowing experiments) and the presence of important small-scale environmental variation not captured with our choice of abiotic variables. Further research is required to clarify the role of seed mass in this diverse annual system, ideally including many focal species, and using sowing experiments.

**Supplementary Information:**

The online version contains supplementary material available at 10.1007/s00442-023-05416-8.

## Introduction

Seed mass is a highly variable and easy-to-measure functional trait, related to the allocation of maternal resources to seeds, with consequences for plant success that start at dispersal. Seed mass underpins a trade-off between seedling survival and seed number (Leishman 2001; Muller-Landau 2010; Turnbull et al. 2004). Heavy-seeded species produce few, well-provisioned seeds that confer high seedling survival and tolerance to harsh abiotic conditions (May et al. 2013; Metz et al. 2010; Moles and Westoby 2004). By contrast, light-seeded species produce many poorly provisioned seeds that are often equipped with physical structures that increase potential dispersal distances. This strategy is thought to increase the probability of some seeds arriving in favourable sites, germinating and rapidly transitioning from reliance on seed provisions to an autotrophic apparatus (DeMalach and Kadmon 2018; Milberg et al. 2000; Rees and Westoby 1997).

In the context of competitive environments, variation in seed mass is theorised to represent a competition-colonization trade-off where heavy-seeded species are superior competitors but weaker colonisers than light-seeded species. However, models representing this strict trade-off require competition to be strongly asymmetric for heavy-seeded species to persist (Rees and Westoby 1997). While competitive hierarchies related to seed mass are commonly reported (Freckleton and Watkinson 2001; Leishman 2001; Tuck et al. 2018; Turnbull et al. 2004) measured competition is not often as asymmetric as models require, and heavy-seeded species are not as rare in nature as these models typically predict (Goldberg and Landa 1991; Muller-Landau 2010). Muller-Landau (2010) instead proposed the tolerance-fecundity trade-off in which the key advantage of large seeds is their superior tolerance of harsh microsites, rather than superior competitive ability. Consistent with this trade-off, the more asymmetric differences in tolerance become, the more diverse the final community (D’Andrea et al. 2013).

The effects of seed mass variation on plant performance and the outcomes of plant-plant interactions can, however, be difficult to isolate. Empirical research on the ecological significance of seed mass is dominated by studies comparing taxa with contrasting life histories (e.g. from trees to small herbs and from long-lived perennials to annuals) (Díaz et al. 2016; Leishman and Westoby 1994; Moles and Westoby 2004; Zepeda and Martorell 2019). In such studies, associations can emerge mainly as a result of shared evolutionary history (e.g., grasses versus other groups) (Godoy et al. 2014; Revell et al. 2008). However, even within the same functional group, seed mass variation is known to be an important driver of community dynamics (Coomes and Grubb 2003). Annuals are well-suited to studies of seed mass effects because they rely exclusively on seeds to regenerate and they are likely to compete as seedlings due to somewhat synchronous germination (Coomes and Grubb 2003; Mathias and Kisdi 2002). In addition, seed mass varies considerably among co-occurring species and has been related to species’ positions along environmental gradients (Dwyer et al. 2015; May et al. 2013; Metz et al. 2010), population size and abundance (Coomes et al. 2002; Guo et al. 2000) and differences in survival (Larson et al. 2019; Metz et al. 2010).

Previous research in annual systems has revealed competitive hierarchies related to seed mass. For example, in a guild of limestone grassland annuals, Turnbull et al. (1999) removed the colonization advantage of light-seeded species by sowing species in equal numbers in both low- and high-density mixtures. They confirmed that heavy-seeded species are recruitment limited and have higher establishment rates than light-seeded species, allowing them to dominate the high-density mixtures (i.e. the mixtures with virtually complete colonisation) (Turnbull et al. 1999). Later work quantifying competitive effects in the same guild confirmed that as neighbour seed size increases (relative to focal plants) so does the competitive effect (Turnbull et al. 2004) and this seed-mass modulated competitive hierarchy persisted even when the guild was transferred to a common garden in a different country (Tuck et al. 2018).

Given these results, it is clear that heavy-seeded neighbours commonly exert strong competitive effects on lighter-seeded focal plants, but the influence of seed mass on species’ responses to plant-plant interactions is less clear. Turnbull et al. (2004) found that heavy-seeded species experienced greater proportional reductions in fecundity due to competition than light-seeded species, despite having the strongest competitive effects. Similarly, Goldberg and Landa (1991) found that species’ competitive effects were more strongly related to seed mass than their competitive responses in a glasshouse study examining the early stages of competition among annuals. In semi-arid grasslands, small-seeded species were more likely to have both strong competitive and facilitative interactions with neighbours, while heavy-seeded species tended to have weak interactions with neighbours (Zepeda and Martorell 2019). However, the outcome of these interactions on the focal’s performance was not estimated (Zepeda and Martorell 2019).

In this study, we investigate the effects of plant-plant interactions on a guild of closely related winter annual species in the Gnaphaliae tribe within the Asteraceae family. The chosen species vary in seed mass by more than an order of magnitude but otherwise have similar erect habits and average biomasses that are not correlated with seed mass. We manipulated natural plant assemblages that included our focal species to determine species’ responses (survival, biomass, seed production) to increasing densities of conspecifics (from zero neighbours to many), and increasing densities of mixed-composition neighbourhoods (including conspecifics and heterospecifics). We address the following research questions:

1. Do species differ in their average responses to density treatments?
2. Does seed mass explain species’ responses to increasing densities of conspecifics?
3. Does seed mass explain species’ responses to increasing densities of both conspecifics and heterospecifics?

## Material and methods

### Study system and species

**Fig. 1:**
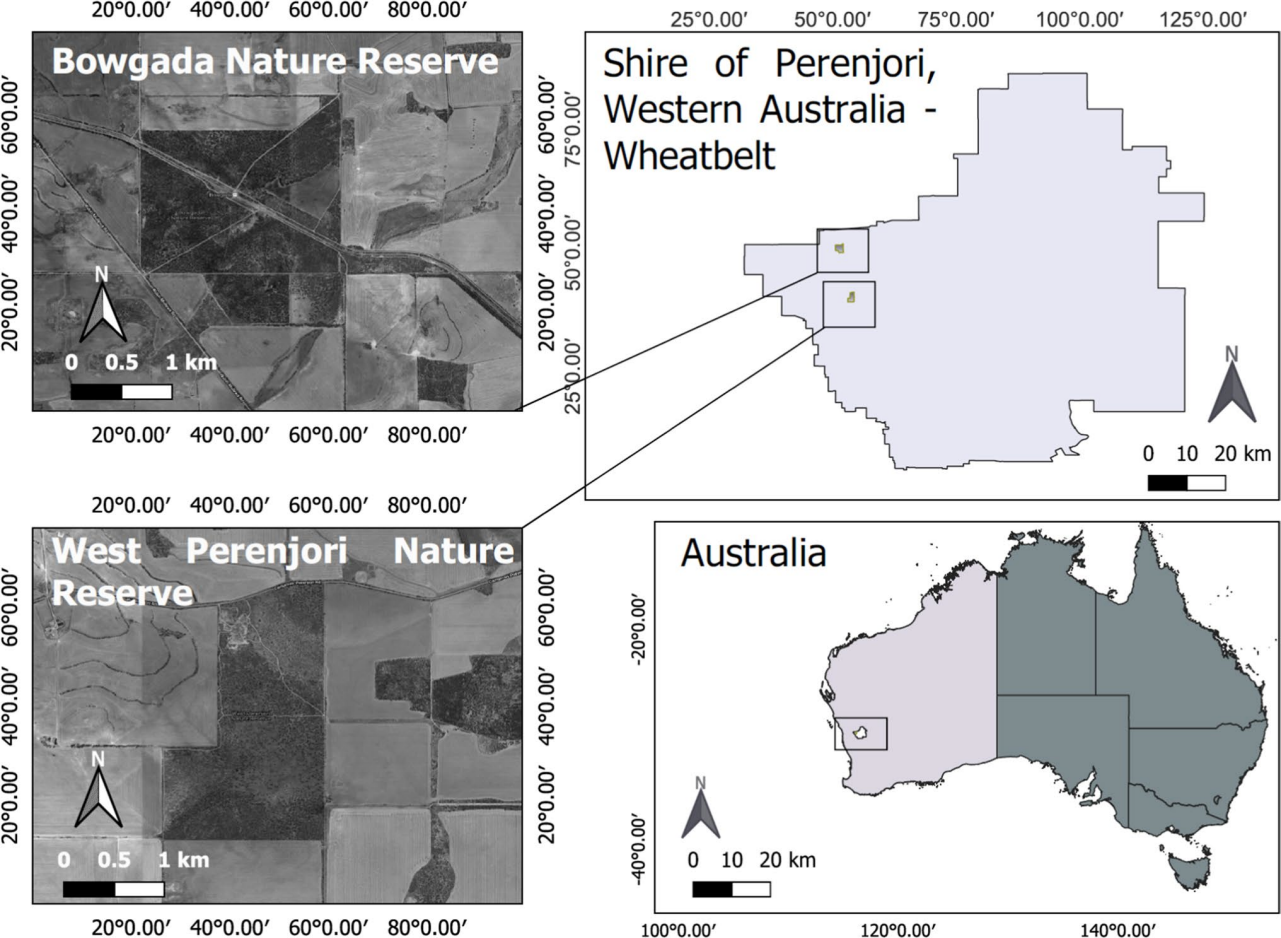
Study area locations. The study was conducted in two close located reserves in Southwest Western Australian Wheatbelt. The state of Western Australia is highlighted in light grey on the bottom right of image

Data for this study were collected between 20-Jul-2020 and 9-Oct-2020 in the York gum-jam woodlands of West Perenjori Nature Reserve ( – 29.46537,116.20908) and Bowgada Nature Reserve (-29.33619, 116.17217) in the Avon Wheatbelt bioregion of Western Australia (Fig. 1). York gum-jam woodlands are found in Mediterranean and semi-arid climates of southwest Western Australia and support diverse annual plant understories. Their sparse, open canopies are dominated by York gum (*Eucalyptus loxophleba*) and jam (*Acacia acuminata*) trees (Prober and Wiehl 2012) (Fig. 1). The annual species in this system typically germinate in June and set seed in September and October. Annual plant assemblages in this location are typically dominated by species from the Asteraceae family, especially members of the Gnaphalieae tribe (Bayer et al. 2002; Jeanes 2021). We selected six common species from this tribe that vary in average seed mass from 0.031 mg to 1.278 mg (Table 1).

**Table 1:** The six focal species, all in the Asteraceae family, in this study, average seed mass (as determined from measurements in this region), maximum densities in Conspecific subplots in the two focal reserves (in a 177 cm^2^ area) and the reserves in which each species was found and included in experimental plots

Species	Seed mass (mg)	Maximum density of neighbours	Location
*Angianthus tomentosus*	0.330*	72	West Perenjori Nature Reserve
*Hyalosperma glutinosum*	0.729	204	West Perenjori Nature Reserve
*Lawrencella rosea*	1.278	29	Bowgada Nature Reserve
*Panaetia lessonii*	0.031	8	West Perenjori Nature Reserve
*Podolepis aristata*	0.123	8	Bowgada Nature Reserve
*Waitzia acuminata*	0.081	12	Bowgada Nature Reserve

*Seed mass values for *Angianthus tomentosus* were sourced from the Kew Garden seed mass database (Kew Royal Botanic 2021)

### Experimental design

Focal species densities were insufficient in each reserve to set up all treatments. Given reserves were close located and share similar climatic conditions we set up experimental plots for each study species in the reserve where that species was most common (Table 1). The use of two reserves allowed for more species to be included in this study rather than serving as a source of replication.

For each species, we setup 12 plots (1 × 1 m) across observed natural gradients of conspecific densities from Jul-2020 to Aug-2020. In each 1 × 1 m plot we established nine circular subplots (15 cm ⌀; 177 cm^2^) centred on an individual focal plant. Once subplots were marked, each subplot was randomly assigned to one of three treatments so that there were three replicate subplots of each treatment in each plot. Treatments were: “Solo”, in which all neighbours were removed around the focal plant; “Conspecific”, the focal plant with all conspecific neighbours kept at the observed natural density and all other neighbours removed; “Mixed” the focal plant with conspecific and heterospecific neighbours retained (Fig. S1). In total, we set up 72 plots, and 648 subplots (and focal individuals), spread evenly across the six focal species. Thinning was undertaken in late June (early in the growing season), when plants were still young, but mature enough that they could be reliably identified. We removed individuals by cutting plants off at the soil level. Individuals did not grow back once cut. Species that form small rosettes at the soil surface were removed with the help of a knife by gently slicing parallel to the soil surface. Only three plots for three different species had densities adjusted by the end of August and the first week or September due to occasional misidentification or mortality of neighbours.

To keep subplot densities relatively constant within plots, Mixed subplots were thinned to approximately match the densities found in the Conspecific subplots for that plot. Thinning in Mixed plots was done arbitrarily with respect to species to prevent biased removal of particular neighbouring species, at the same time targeting the removal of conspecifics for focal species in which the proportion of conspecifics was markedly higher than heterospecifics, such as for *Hyalosperma glutinosum*. In September 2020, for Mixed subplots, we identified all neighbour species and recorded their abundances. Density was also re-calculated to capture the number of potentially interacting plants during the vegetative life stage. The highest neighbour densities in Conspecific plots recorded for each species are shown in Table 1.

### Performance and environmental variables

For each of the 216 focal individuals we recorded survival and reproductive output, and estimated biomass (mg) at peak flowering using species-specific allometric equations (Table S1, Supplementary Methods) to avoid destructive measurements. One of the focal species, *Angianthus tomentosus*, did not set seed in time for data collection and thus biomass (mg) was measured directly on focal individuals, except for three individuals whose samples were lost. Survival was determined at the end of the season by assigning 1 to individuals that attempted reproduction and 0 to those that died prior to flowering. To limit error in seed set counts, we placed organza bags over pollinated flowers to ensure any seeds that dropped early were captured and included in seed counts. For *A. tomentosus* we used the number of inflorescences as a proxy for reproductive effort given that seed set did not occur in time for direct seed counting.

To describe variation in the local environment of plots, we assessed soil chemistry, soil texture, and the cover of tree canopies and bare soil in each 1 × 1 m plot. We analysed soil chemistry from composite soil samples, obtained by collecting soil in the 0–20 cm layer from three different positions immediately adjacent to the plot. We did not sample soils in plots as this was too disruptive of the focal plants. Once collected in clean plastic containers, we air-dried, and sieved (2 mm mesh) soil samples and sent them for analysis (School of Agriculture and Food Sciences, The University of Queensland) of nitrate, potassium (K^+^), calcium (Ca^2+^), magnesium (Mg^2+^) and aluminium (Al^3+^). Next, we calculated cation exchange capacity (CEC) by summing all the cations (K^+^, Ca^2+^, Mg^2+^ and Al^3+^). To quantify soil texture we used a particle size analyser at the Chemistry Centre of the Queensland Government Department of Environment and Science. We obtained percentages of particle sizes for coarse and fine sand, silt and clay. We estimated tree canopy cover using a spherical densiometer, with measurements taken in the four cardinal directions of each plot and then averaged. To estimate the proportion of bare soil in each plot we traced areas of bare soil using photos of each plot imported into ImageJ (Schneider et al. 2012).

Prior to statistical modelling, we performed a principal component analyses (PCA) on the measured environmental variables (CEC, nitrate (mg/Kg), clay (%), sand (%), canopy cover (%) and bare soil (%) to generate two orthogonal axes of environmental variation (Table S2). All environmental variables were log-transformed prior to fitting the PCA except for canopy cover, bare soil and sand. PCA axis 1 (PC1) described a gradient from plots with high canopy cover, high sand content and low CEC to plots with high clay and high bare soil percentages and high CEC (Table S2). PCA axis 2 (PC2) described a gradient from low to high nitrate levels (Table S2, Fig. S2). Environment PC1 also represented a difference in the environment of our studied sites, where West Perenjori Nature Reserve was characterized by more sun-exposed areas and higher soil fertility than Bowgada Nature Reserve (Fig. S2).

### Statistical modelling

All analyses were performed in R 4.3.0 (R Foundation for Statistical Computing 2018). Focal plant performance was assessed using survival, adult biomass and reproductive output. In the models used to address each research question, all six species were included to enable comparisons within and among species. For each species, only one average seed mass value was considered (Table 1). Survival was modelled as a binary response using a binomial distribution and logit link function. Biomass was relativised as the percentage of the maximum log-transformed biomass for each species, hereafter referred to as “relative log(biomass)”, to account for intrinsic differences in biomass among species. This response was modelled using a Gaussian distribution. To facilitate comparison across species that differ in fecundity (due to variation in seed mass), reproductive output was also relativised as the percentage of the maximum reproductive effort for each species. This relative fecundity response was expressed as an integer and modelled using a negative binomial distribution and log link function. Mixed-effects models were used to account for observations nested within plots, and observations nested within species. Specifically, we used the packages lme4 (Bates et al. 2021) and glmmTMB (Magnusson et al. 2021). All continuous explanatory variables were standardized to zero mean and unit variance before analyses.

To address question 1 regarding differences among species in their average responses to the density treatments, we fitted models for each performance measure with species, density treatment (Solo, Conspecific or Mixed) and their two-way interaction as fixed effects, and plot as a random effect. We also allowed treatment effects to vary by plot in the random effects structure. After fitting this model, we used emmeans() function from the emmeans package (Lenth et al. 2021) to estimate marginal means and conducted tests for differences within species and joint tests for differences among species.

To address question 2, regarding the role of seed mass for explaining species’ responses to increasing densities of conspecifics we subsetted the data to include only Solo and Conspecific focal plants. We fitted mixed-effects models for each performance measure as a function of conspecific density (linear and quadratic terms), seed mass, and two-way interactions between seed mass and the density terms. We log-transformed conspecific density to log(density + 1) to improve linearity with the various responses. We included quadratic density terms to test for possible hump-shaped responses indicating facilitation at moderate neighbour densities (Dickie et al. 2005; Zhang and Tielbörger 2020). We also included environmental PCA axes as covariates to capture variation in performance not explained by neighbour density. Plot and species were included as random effects. We allowed species to have their own relationships with conspecific density (linear and quadratic). We also fitted single-species models for each performance variable with log(conspecific density + 1) and the environmental covariates (PC1 and PC2) as main effects. Quadratic terms were also included for conspecific density where significant. The plot was included as a random effect.

To address question 3 regarding the role of seed mass in modulating species’ responses to increasing densities of both conspecifics and heterospecifics, we subsetted the data to include only Solo and Mixed focal plants. We fitted the same fixed and random effects as in the models for conspecifics only, with the addition of log-transformed heterospecific density (linear and quadratic) and interactions between seed mass and heterospecific density terms. We also allowed species to vary in their relationships with both conspecific and heterospecific density (linear only) in the species-level random effects structure. Quadratic terms were excluded from the random effects to prevent overfitting.

We used the tab_model() function from sjPlot package (Lüdecke 2021) to extract model summaries. The conditional and marginal coefficient of determination (R^2^) were calculated using the r.squaredGLMM() function from the MuMIn (Bartoń, 2020). For survival, we reported R^2^ as the “theoretical” estimation and the “lognormal” estimation for relative reproductive output.

## Results

1. Do species differ in their average responses to density treatments?
  For the survival response, the interaction between species and density treatment was not significant (Table 2), nor was the main effect for density treatment. Inspection of estimated marginal means (Fig. 2) revealed that *L. rosea* was the only species that exhibited a significant survival response to density treatment, with Solo plants having a higher survival probability than plants growing in the Mixed treatment (*P* = 0.046) (Fig. 2; Table S4). The interaction between species and density treatment was significant for the remaining responses (Table 2). For relative log(biomass), the interaction captured different density responses between *A. tomentosus*, *P. aristata* and *P. lessonii*. For *A. tomentosus*, Solo plants produced significantly more biomass than plants in Conspecific (*P* = 0.043) or Mixed treatments (*P* = 0.007), whereas for *P. aristata* and *P. lessonii* Solo plants had lower biomass than in Conspecific or Mixed treatments, but differences were only significant for the Mixed treatments (*P* = 0.033 and *P* = 0.006, respectively) (Fig. 2; Table S4). For reproductive output, the significant interaction captured differential density responses between *A. tomentosus* and *L. rosea*. Solo plants of *A. tomentosus* produced significantly more inflorescences than plants in Conspecific (*P* = 0.034) and Mixed treatments (*P* = 0.006) (Fig. 2; Table S4), whereas for *L. rosea*, Solo plants produced significantly fewer seeds than plants in Conspecific (*P* < 0.001) or Mixed treatments (*P* = 0.003).

**Table 2:** ANOVA summary tables for models containing species, treatment and their interaction for the three response variables

*Predictors/Model term*	df1	df2	F.ratio	*P* value
Probability of survival				
*Species*	5	NA	4.733	** < 0.001**
*Treatment*	2	NA	0.157	0.855
*Species:Treatment*	10	NA	1.007	0.434
Rel. log(Biomass)				
*Species*	5	66.13	27.517	** < 0.001**
*Treatment*	2	64.13	2.269	0.304
*Species:Treatment*	10	63.53	1.774	**0.013**
Rel. reproductive output				
*Species*	5	510.00	2.839	**0.015**
*Treatment*	2	510.00	0.155	0.857
*Species:Treatment*	10	510.00	3.424	** < 0.001**

Significant *P-values* (*P* < 0.05) are in bold

Treatments were Solo (neighbours removed), Conspecific (conspecific neighbours only) and Mixed (heterospecific and conspecific neighbours)

**Fig. 2:**
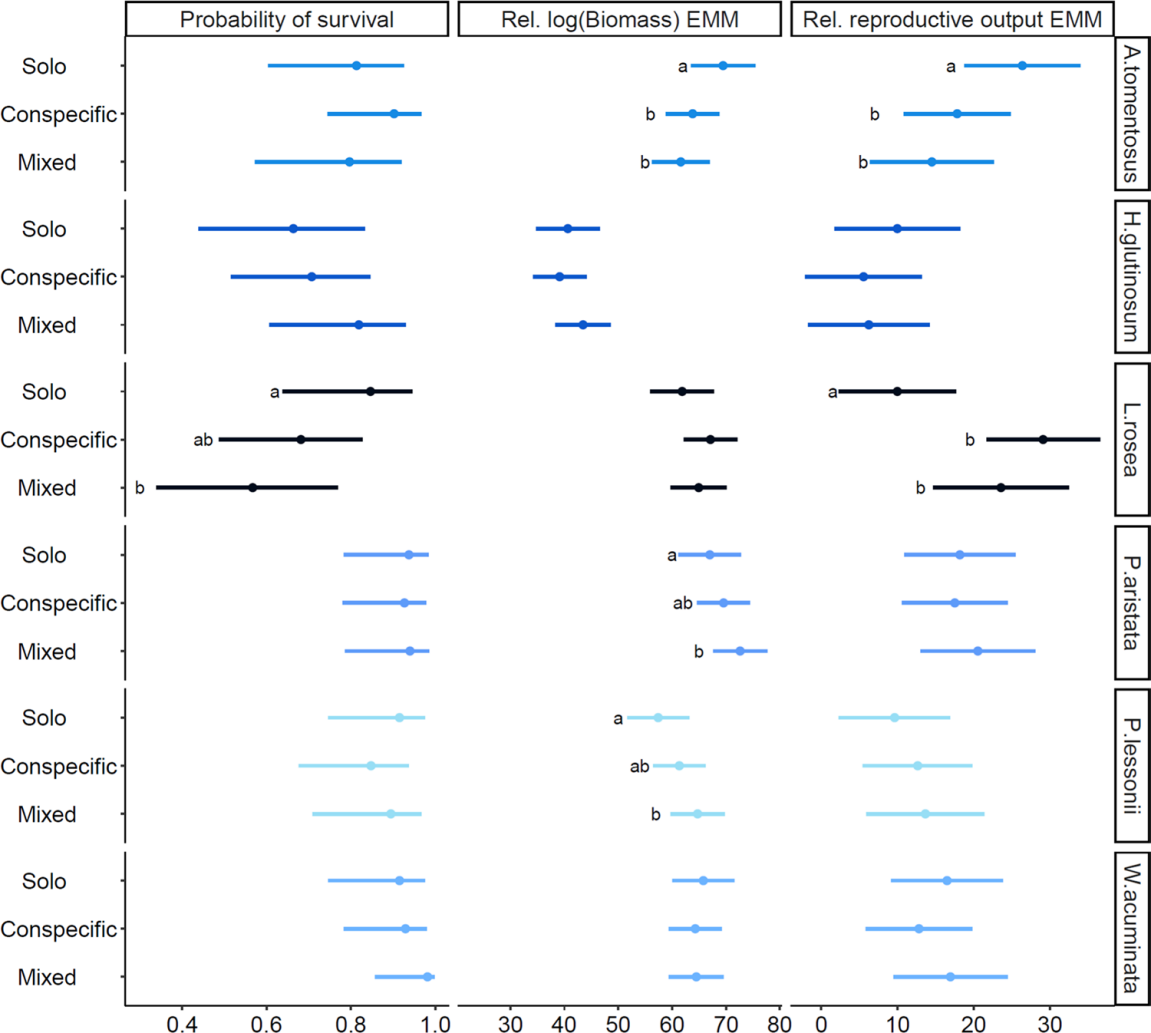
Estimated marginal means (EMMs) and probabilities for the six winter annuals in this study: *Angianthus tomentosus, Hyalosperma glutinosum, Lawrencella rosea, Panaetia lessonii, Podolepis aristate, Waitzia acuminata*. All estimates are on the original scale (back transformed). Lines correspond to 95% confidence intervals and points are EMMs for biomass and relative reproductive output. For survival, points are probabilities (Table S3). Treatments were Solo (neighbours removed), Conspecific (conspecific neighbours only) and Mixed (heterospecific and conspecific neighbours). Different letters represent significant differences between the treatments within a species and response variable. Contrasts and associated *P-values* can be found in Table S4
2. Does seed mass explain species’ responses to increasing densities of conspecifics?
  For the models containing only Solo and Conspecific treatments, biomass was not related to any of the fixed explanatory variables. The probability of survival was explained solely by a negative relationship with seed mass (Fig. 3a; Table 3), though the explanatory power of this model was low (marginal R^2^ = 0.11, Table 3). Environment PC1 was negatively related to relative reproductive output, such that plants in shady and sandy areas had higher relative reproductive output than plants growing in sun-exposed and more fertile areas (Fig. 3b; Table 3). However, most species did not occur along this entire gradient, and the fitted relationship was driven in part by the low relative reproductive output values of *H. glutinosum* that occurred mostly in the sun-exposed part of environment PC1 (Fig. 3b). Again, the amount of variance explained by fixed effects was low (marginal R^2^ = 0.17). Conspecific density was not significant in any of these models. Single species models examining conspecific density effects showed that only *H. glutinosum* (marginal R^2^ = 0.16) and *L. rosea* (marginal R^2^ = 0.35) had reproductive output significantly reduced by increasing densities of conspecifics (Fig. 4; Table S5–S7).

**Fig. 3:**
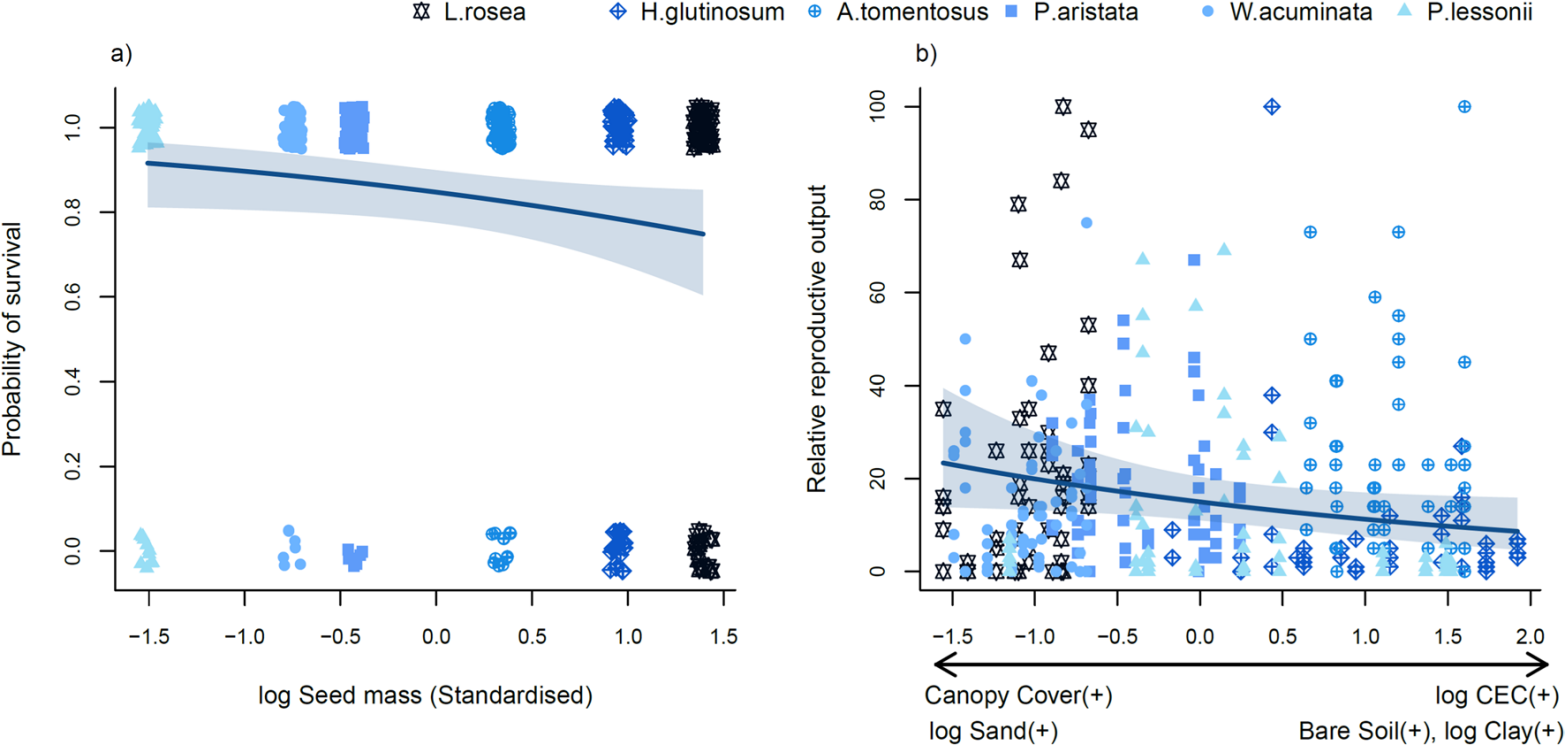
Bivariate plots from final multilevel regression models for plots containing conspecifics. **a** Shows the relationship between the probability of survival and seed mass. **b** Shows the relationship between relative reproductive output and environment PC1. Shaded areas represent ± 95% confidence intervals. Points in “a” were jittered to show overlapping values more clearly. Colours for species run from the darkest/heaviest (*L. rosea* = 1.278 mg) to the lightest (*P. lessonii* = 0.031 mg)

**Table 3:** Final multilevel model estimates for plots containing only conspecifics

Predictors	Probability of survival			Rel. log (Biomass)			Rel. reproductive output		
	Estimate	CI (95%)	*P*-value	Estimate	CI (95%)	*P*-value	Estimate	CI (95%)	*P*-value
Intercept	1.72	1.24 – 2.19	** < 0.001**	62.55	52.75 – 72.36	** < 0.001**	2.71	2.40 – 3.02	** < 0.001**
std_log(Conspecific density)	0.22	– 0.21 to 0.65	0.315	1.11	– 1.67 to 3.89	0.433	0.05	– 0.23 to 0.34	0.719
std_log(Conspecific density^2)	0.22	– 0.31 to 0.75	0.413	– 2.01	– 5.78 to 1.76	0.295	– 0.34	– 0.84 to 0.17	0.19
std_Environment PC1	– 0.26	– 0.58 to 0.05	0.098	– 1.31	– 4.81 to 2.20	0.464	– 0.29	– 0.56 to – 0.01	**0.041**
std_Environment PC2	0.22	– 0.11 to 0.54	0.188	0.31	– 1.65 to 2.27	0.756	0.07	– 0.12 – 0.26	0.475
std_log(Seed mass)	– 0.45	– 0.90 to – 0.00	**0.049**	– 1.22	– 11.06 to 8.62	0.808	0.21	– 0.09 to 0.51	0.164
std_log(Conspecific density)*sdt_log(Seed mass)	– 0.18	– 0.61 – 0.25	0.412	– 0.07	– 2.83 to 2.68	0.958	0.25	– 0.05 to 0.56	0.102
std_log(Conspecific density^2)*sdt_log(Seed mass)	– 0.35	– 0.89 to 0.20	0.218	– 2.22	– 6.39 to 1.94	0.295	– 0.04	– 0.55 to 0.47	0.891
Random Effects				97.90			0.7		
σ^2^	3.29			45.90 _plot:species_			0.37 _plot:species_		
τ_00_	0.32 _plot:species_			140.76 _species_			0.02 _species_		
	0.00 _species_			7.32 _species.std_log_dens_			0.04 _species.std_log_ Conspecicific density_		
τ_11_	0.00 _species.std_log_Conspecicific density_			13.42 _species.I(std_log_dens^2)_			0.07 _species.I(std_log_ Conspecicific density ^2)_		
	0.00 _species.I(std_log_Conspecific density^2)_			0.18 _species.std_log_dens_			– 0.95 _species.std_log_ Conspecicific density_		
ρ_01_				– 0.62 _species.I(std_log_dens^2)_			0.95 _species.I(std_log_ Conspecicific density ^2)_		
Observations	432			389			359		
Marginal R^2^ / Conditional R^2^	0.11/0.19			0.13/0.68			0.17/0.55		

CI = confidence interval. Significant effects (P < 0.05) are in bold

**Fig. 4:**
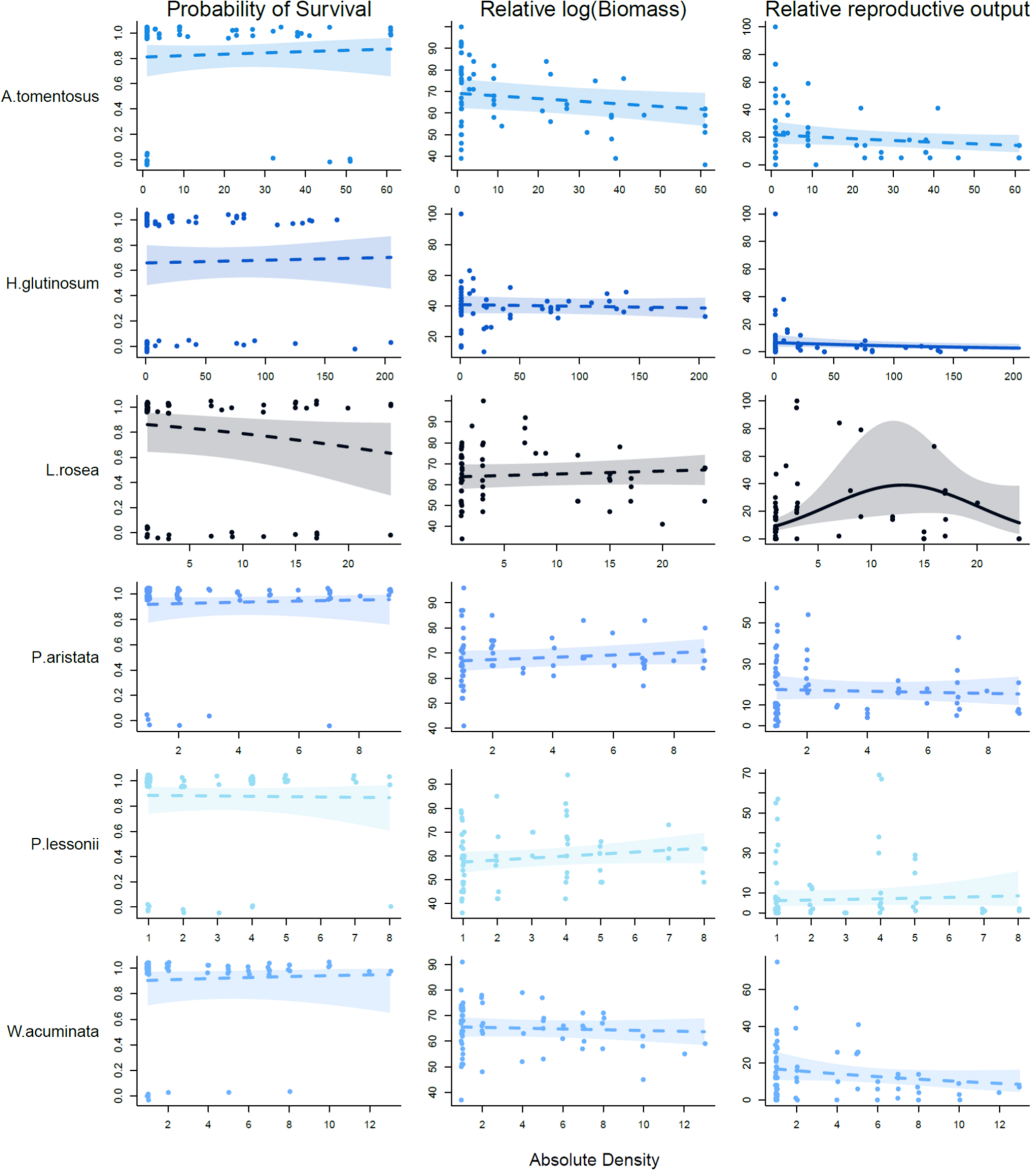
Bivariate plots from single-species regression models for plots containing conspecifics. Each column corresponds to the performance variable on the Y axis, the X axis is given in absolute density and species are given on rows. Shaded areas represent ± 95% confidence intervals. Points were jittered to show overlapping values more clearly. Plots with solid lines show significant effects in response to increasing densities of conspecifics (*P* < 0.05)
3. Does seed mass explain species’ responses to increasing densities of both conspecifics and heterospecifics?
  For models containing Solo plants and plants from Mixed neighbourhoods, relative reproductive output (R^2^ = 0.10) was not related to any of the fixed explanatory variables. In the relative log(biomass) model (also R^2^ = 0.10), the interaction between seed mass and the squared term for heterospecific density was marginally significant (*P* = 0.041, Table 4) and indicated that light-seeded species exhibited a shallow hump-shaped relationship with heterospecific density whereas heavy-seeded species exhibited a shallow U-shaped relationship (Fig. S3), but given the uncertainty surrounding this interaction, it is unlikely to be biologically significant.

**Table 4:** Final multilevel model estimates for plots containing both conspecifics and heterospecifics (Mixed)

Predictors	Survival			Rel. log(Biomass)			Rel. reproductive output		
	Estimate	CI (95%)	*P*-value	Estimate	CI (95%)	*P*-value	Estimate	CI (95%)	*P*-value
Intercept	1.89	1.23 – 2.55	** < 0.001**	61.20	51.87 – 70.53	** < 0.001**	2.57	2.20 – 2.94	** < 0.001**
std_log(Conspecific density)	0.82	– 0.26 to 1.90	0.137	– 0.55	– 5.62 to 4.52	0.831	– 0.14	– 0.56 to 0.27	0.504
std_log(Conspecific density)^2	– 0.47	– 1.09 to 0.16	0.142	– 1.05	– 3.79 to 1.70	0.454	– 0.04	– 0.32 to 0.25	0.806
std_log(Heterospecific density)	– 0.39	– 0.94 to 0.16	0.163	0.59	– 2.58 to 3.76	0.716	0.18	– 0.07 to 0.42	0.161
std_log(Heterospecific density)^2	0.22	– 0.25 to 0.69	0.363	– 0.30	– 2.34 to 1.74	0.772	– 0.03	– 0.24 to 0.18	0.785
std_Environment PC1	– 0.3	– 0.66 to 0.07	0.108	0.55	– 2.94 to 4.03	0.757	– 0.16	– 0.42 to 0.10	0.218
std_Environment PC2	0.29	– 0.09 to 0.66	0.132	0.77	– 1.25 to 2.79	0.455	0.13	– 0.05 to 0.31	0.165
std_log(Seed mass)	– 0.55	– 1.24 to 0.13	0.114	– 4.57	– 13.97 to 4.83	0.339	0.05	– 0.32 to 0.42	0.785
std_log(Conspecific density)*sdt_log(Seed mass)	1.06	0.01 – 2.10	**0.048**	3.15	– 1.83 to 8.14	0.214	0.1	– 0.32 to 0.51	0.651
std_log(Conspecific density)^2*sdt_log(Seed mass)	0.07	– 0.54 to 0.68	0.817	– 1.24	– 4.09 to 1.62	0.395	– 0.09	– 0.39 to 0.21	0.547
std_log(Heterospecific density)*std_log(Seed mass)	– 0.68	– 1.19 to – 0.16	**0.01**	– 2.15	– 5.19 to 0.88	0.164	0.05	– 0.19 to 0.30	0.662
std_log(Heterospecific density)^2*std_log(Seed mass)	– 0.16	– 0.71 to 0.40	0.584	2.46	0.10 – 4.81	**0.041**	0.06	– 0.20 to 0.32	0.663
Random Effects		3.29			106.46			0.7	
σ^2^		0.69 _plot:species_			46.46 _plot:species_			0.34 _plot:species_	
τ_00_		0.00 _species_			123.25 _species_			0.08 _species_	
τ_11_		0.00 _species.std_log_Conspecific density_			16.29 _species.std_log_Conspecific density_			0.00 _species.std_log_Conspecific density_	
		0.00 _species.std_log_Heterospecific density_			7.76 _species.std_log_Heterospecific density_			0.01 _species.std_log_Heterospecific density_	
ρ_01_		– 1.00 _species.std_log_Conspecific density_			– 1.00 _species.std_log_Conspecific density_			0.93 _species.std_log_Conspecific density_	
		1.00 _species.std_log_Heterospecific density_			0.91 _species.std_log_Heterospecific density_	–		1.00 _species.std_log_Heterospecific density_	
Observations		432			384			355	
Marginal R^2^ / Conditional R^2^		0.18/0.32			0.10/0.66			0.10/0.45	

**Fig. 5:**
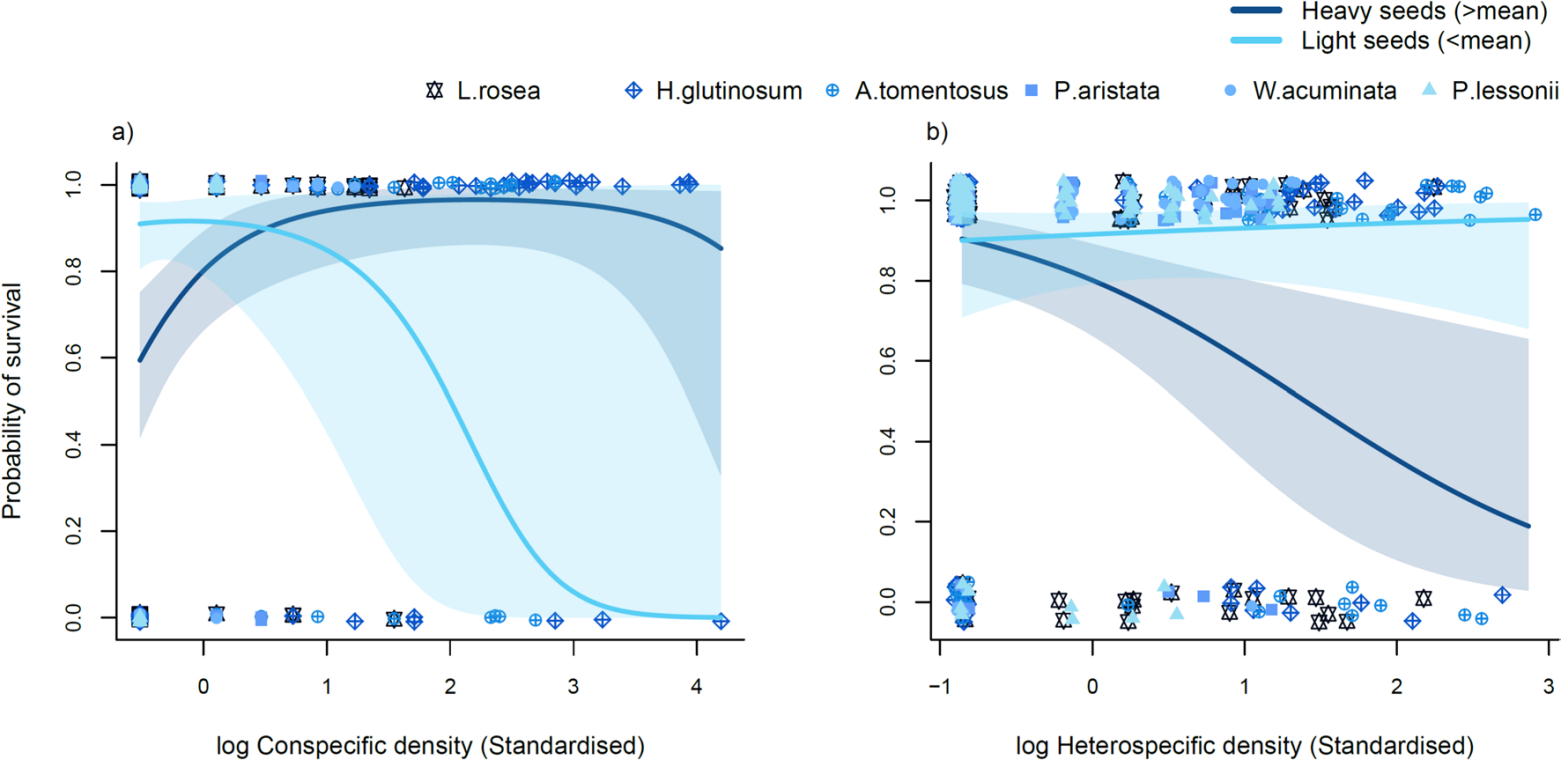
Bivariate plots from final multilevel regression models for plots containing mixtures of conspecifics and heterospecifics (Mixed). **a** Shows the relationship between the probability of survival and increasing densities of conspecifics with separate fitted lines for light-seeded and heavy-seeded species. **b** Shows the relationship between the probability of survival and increasing densities of heterospecifics with separate fitted lines for light-seeded and heavy-seeded species. Points in “**a**” and “**b**” were jittered to show overlapping values more clearly. Shaded areas in both plots represent ± 95% confidence intervals. Colours for species run from the darkest/heaviest (*L. rosea* = 1.278 mg) to the lightest (*P. lessonii* = 0.031 mg)

*CI*  confidence interval. Significant effects (*P* < 0.05) are in bold
  For the probability of survival (R^2^ = 0.18), the interaction between seed mass and conspecific density was marginally significant (*P* = 0.048, Table 4), and indicated that survival of heavy-seeded species increased with conspecific density, while survival of light-seeded species declined sharply with increasing conspecific density (Fig. 5a), though confidence intervals were large indicating considerable uncertainty. The interaction between seed mass and heterospecific density was also significant (*P* = 0.01, Table 4) and showed the opposite trend. Light-seeded species had high survival probabilities regardless of heterospecific density, while heavy-seeded species experienced declines in survival with increasing heterospecific density (Fig. 5b).

## Discussion

For six closely related annual plant species we found limited evidence that neighbour density affected performance. When the density treatment was treated as a factor to assess overall treatment effects, some species exhibited significant differences between treatments for certain responses (6 of the 18 species × response combinations). When neighbour densities were modelled as continuous variables, the Mixed-assemblage model for survival was the only model to indicate density effects on performance. In this model, seed mass interacted with both conspecific density and heterospecific density to affect survival in opposing ways, though the relationships were highly uncertain. Seed mass was also directly related to the probability of survival in the model containing only conspecifics, and counter to expectations this relationship was negative. This study, therefore, highlights the uncertain role of seed mass in modulating plant-plant interactions among closely related annual species.

### Weak evidence for plant-plant interactions

In models of overall density treatment effects, only 3/18 species × response combinations indicated competitive effects (i.e. highest performance in Solo plants; Fig. 2) and in neither case was the Conspecific density treatment different from the Mixed density treatment. When neighbour densities were treated as continuous variables in linear mixed-effects models, effects of density were only evident in Mixed assemblages containing both conspecifics and heterospecifics and only for survival. Conspecific density was negatively related to survival, but only for light-seeded species, whereas heterospecific density was negatively related to survival for heavy-seeded species (though fitted relationships were highly uncertain). Competition with conspecifics is considered one of the most important mechanisms leading to coexistence in communities (Chesson 2000) and has been found to be stronger than competition with heterospecifics in many ecosystems (Adler et al. 2018; Tuck et al. 2018; Turnbull et al. 1999); this is, however, not strongly consistent with our results and previous research in our study system. For instance, Towers et al. (2020) found that only 6 of their 11 focal species exhibited significant negative relationships with conspecific density and attributed some of the non-significant results to low densities of some species in natural assemblages. Similarly, conspecific densities for two of our studied species, *P. aristata* and *P. lessonii* only reached maximums of 8 plants per 177 cm^2^. Even our highest-density species (*H. glutinossum*) only reached 204 individuals per 177 cm^2^ (1.15 individuals per cm^2^) which is low compared with previous competition experiments. For instance, Tuck et al. (2018) achieved conspecific densities of 3.5 individuals per cm^2^ and Turnbull et al., (1999) sowed higher densities (20 seeds per cm^2^ in the highest density treatment) than the natural seed rain (less than 1 seed per cm^2^) to remove colonisation advantages. For some species, high densities may not be observed in natural conditions and mechanisms other than competition with conspecifics may be operating. It is also possible that negative density dependence occurred at earlier life stages than we examined, notably during germination and seedling establishment. These early life stages are known to have important density-dependent regulatory effects in communities of annual species (Tielbörger and Rüdiger 2009). For example, high densities of seeds can result in delayed germination or reduce the fraction of seeds that germinate in a given season, buffering competition among germinants in some annual systems (Angert et al. 2009; Gremer and Venable 2014). Future research would benefit from understanding how changes in the density of neighbours affect performance across life stages within and between years in this and other communities.

Another possible explanation for the weak evidence for plant-plant interactions observed in this study is counteracting negative and positive interactions (Zepeda and Martorell 2019). Indeed, we found evidence of both positive interactions (i.e. low performance in Solo plants, Fig. 2) and negative interactions (i.e. high performance in Solo plants, Fig. 2) in models of overall density treatment effects. Also, in the same York gum woodland study system, Bimler et al. (2018) found that positive interactions were common among conspecifics and heterospecifics and that the direction of interactions can switch depending on the abiotic context. The direction of interactions may also switch depending on the identity of neighbouring plants (Zepeda and Martorell 2019), which was not considered in the present study beyond the separation of conspecific neighbours from heterospecific neighbours.

### Seed mass effects

The strongest evidence that seed mass influenced performance, both overall and in the context of plant-plant interactions, was found in our models for survival. Overall, seed mass was negatively related to survival in the model including Solo and Conspecific plants. This negative result was counter to theoretical expectations (Muller-Landau 2010) and empirical evidence from annual systems (Metz et al. 2010) and other systems more generally (Moles and Westoby 2006). One reason for this discrepancy may be that most studies revealing positive relationships include a much wider range of seed mass values from species with substantially different life history strategies, rather than a gradient among closely related species like examined here (Coomes and Grubb 2003). Alternatively, the effects of seed mass on survival have been previously linked to the sensitivity of light-seeded species to inter-annual variation in precipitation and temperature compared to heavy-seeded species (Coomes and Grubb 2003; Metz et al. 2010). As such, the negative relationship we observed may have been driven by specific weather conditions during the study period that favoured light-seeded species. Inter-annual variation is a potential mechanism of diversity maintenance for species differing in seed mass and should be investigated in the future (Metz et al. 2010; Pake and Venable 1996). Our results may also reflect an unmeasured relationship between seed mass and time to germination (Coomes and Grubb 2003; Huang et al. 2016). If light-seeded species germinate earlier than heavy-seeded species, they are more likely to experience high survival (Verdú and Traveset 2005; Wainwright et al. 2012), though this relationship remains to be tested. It is also possible that we simply did not have enough species to reliably test seed mass effects in this system. For example, the low survival probabilities observed for our heaviest-seeded species (*L. rosea*) were very influential in the observed negative relationship between seed mass and survival (Fig. 3a). Without more heavy-seeded species to include it is not possible to determine how general this observation is.

Though some of our results were unexpected, our study is also not alone in deviating from theorized expectations. For instance, in other Mediterranean-climate systems, light-seeded annual species seem to thrive in harsher conditions than heavy-seeded ones (Harel et al. 2011; May et al. 2013). Light-seeded species may experience weaker competition than heavy-seeded species, or they may even be routinely facilitated. For example, we found neutral effects of heterospecific density on survival for light-seeded species and strong competitive effects on heavy-seeded species (Fig. 5b). Although the opposite was true for increasing densities of conspecifics. However, these results must be interpreted with caution given that focal species differed in the maximum densities they experienced.

### Limitations

In natural systems, species’ responses to neighbours are fundamentally mediated by the environment and rigorous explanations of diversity patterns need to account for environmental heterogeneity (Bimler et al. 2018; Sears and Chesson 2007; Towers et al. 2020). In this study of natural assemblages, species had presumably been ‘pre-filtered’ into different microsites within the measured environment (Fig. 3b and S2). The lack of orthogonality between seed mass and environmental variables meant that we could not examine if seed mass modulates the outcomes of plant-plant interactions along environmental gradients. Instead, environmental variables were included as covariates to capture what could not be explained by seed mass and neighbour densities. Our results indicated that environmental differences between reserves, rather than small-scale environmental heterogeneity, were most related to species’ performance. Species located in Bowgada Nature Reserve had higher relative reproductive output compared with species in West Perenjori Nature Reserve but we have no way to separate species effects from location effects.

A close inspection of our models showed that most of the variation not explained by fixed effects occurred between plots and between species. This unexplained variation, together with the fact that species occurred in different parts of the environment, indicate that differences in species’ performance were likely associated with small-scale environmental variation, even though we could not detect it with our choice of abiotic and biotic variables. Additional variables likely to affect species responses at small spatial scales include interactions with soil biota (Teste et al. 2017), and tree exudates (Hobbs and Atkins 1991), among others. Future research should consider expanding the investigated biotic and abiotic variables to capture small-scale variation in species’ responses that remained unexplained in this study.

The contradictory weak evidence of competition with conspecifics observed in this study could have been at least partially caused by the experimental thinning itself. In this case, thinning could have disturbed plots to the point that Solo plants had their performance impacted more by thinning than focal plants in other treatments (Conspecific and Mixed neighbourhoods). However, thinning was done early in the life stage when root systems were not fully developed, and care was taken to remove only aboveground structures, so we are confident that such thinning effects were minimal.

## Conclusion

Using natural assemblages of annuals we investigated the potential of seed mass to explain species’ differential responses to neighbour density. By limiting the scope of the study to the same life history strategy, and closely related species therein, we aimed to minimise variation in species’ strategies other than their differences in seed mass. Within this guild of closely related annuals, our results were weak overall, and where significant, they were often counter to theoretical and empirical expectations. First, we found limited evidence of competition and no evidence that intraspecific competition was stronger than interspecific competition, though we cannot rule out that negative density dependence occurred at earlier life stages than we examined. Second, we found a negative relationship between seed mass and overall survival. This result may have arisen by chance given that we only included six focal species or may reflect an advantage for light-seeded species during the specific growing season studied. Finally, we revealed some evidence that seed mass modulates species’ survival responses to increasing densities of both conspecifics and heterospecifics. Survival of light-seeded species appeared to be more affected by conspecific density than heavy-seeded species, whereas the opposite was true in response to heterospecific density. Future research should include more focal species to increase the power of analyses involving seed mass. In addition, sowing experiments would allow for all species to be sown along entire environmental gradients and could also be used to create similar ranges of neighbour densities across species. We also recommend measuring other environment variables likely to affect species performance at a plot level, such as soil microbial composition and diversity. Finally, the possible effects of seed mass on the outcomes of plant-plant interactions should also be examined at the germination and early seedling stages.

## Supplementary Information

Below is the link to the electronic supplementary material.

Supplementary file1 (DOCX 3274 KB)

## Data Availability

The data for this study is available on Dryad.

## Code availability

The data and code for this study is available on Dryad 10.5061/dryad.573n5tbb3.
