# Supplementary material for: Weak evidence of trade-offs modulated by seed mass among a guild of closely related winter annuals
Source: Oecologia. 2023 Jul 12;202(3):561–75. doi: 10.1007/s00442-023-05416-8 (PMC10386915; doi:10.1007/s00442-023-05416-8)
Supplement: Supplementary file 1 — Supplementary file1 (DOCX 3274 KB) [file 442_2023_5416_MOESM1_ESM.docx]

**Oecologia**

**Title:** Weak evidence of trade-offs modulated by seed mass among a guild of closely related winter annuals

**Authors:** Isis A. da Silva^1^, Margaret M. Mayfield^2^ and John M. Dwyer^1^

Corresponding author: Isis A. da Silva

i.arenddasilva@uqconnect.edu.au

^1^School of Biological Sciences, The University of Queensland, St Lucia, QLD, Australia

^2^ School of Biological Sciences, The University of Melbourne, Parkville, VIC, Australia

Supplementary Methods

Parameterising biomass allometric equations

For the allometric equations (Table S1) we built different linear models for each species using the lm function in R. The response was individual biomass (mg) measurements from 8 to 11 individuals across all species. The models included the total number of individuals collected across all 12 plots. Explanatory variables were height, number of flowers (or flower buds or flowers plus buds, depending on the species), number of leaves, number of stems. Number of leaves were measured only for *Podolepis aristata* and *Panaetia lessonii.* We selected the fixed effects based on correlations among predictors. Biomass and height were logged for all models. We inspected model fits and if necessary removed occasional extreme biomass values. In sequence we extracted significant coefficients for building our equations.

For *Angianthus tomentosus* predicted biomass was used only for the individuals in plot 45, subplot M2 and C2, and plot 410, subplot S1.


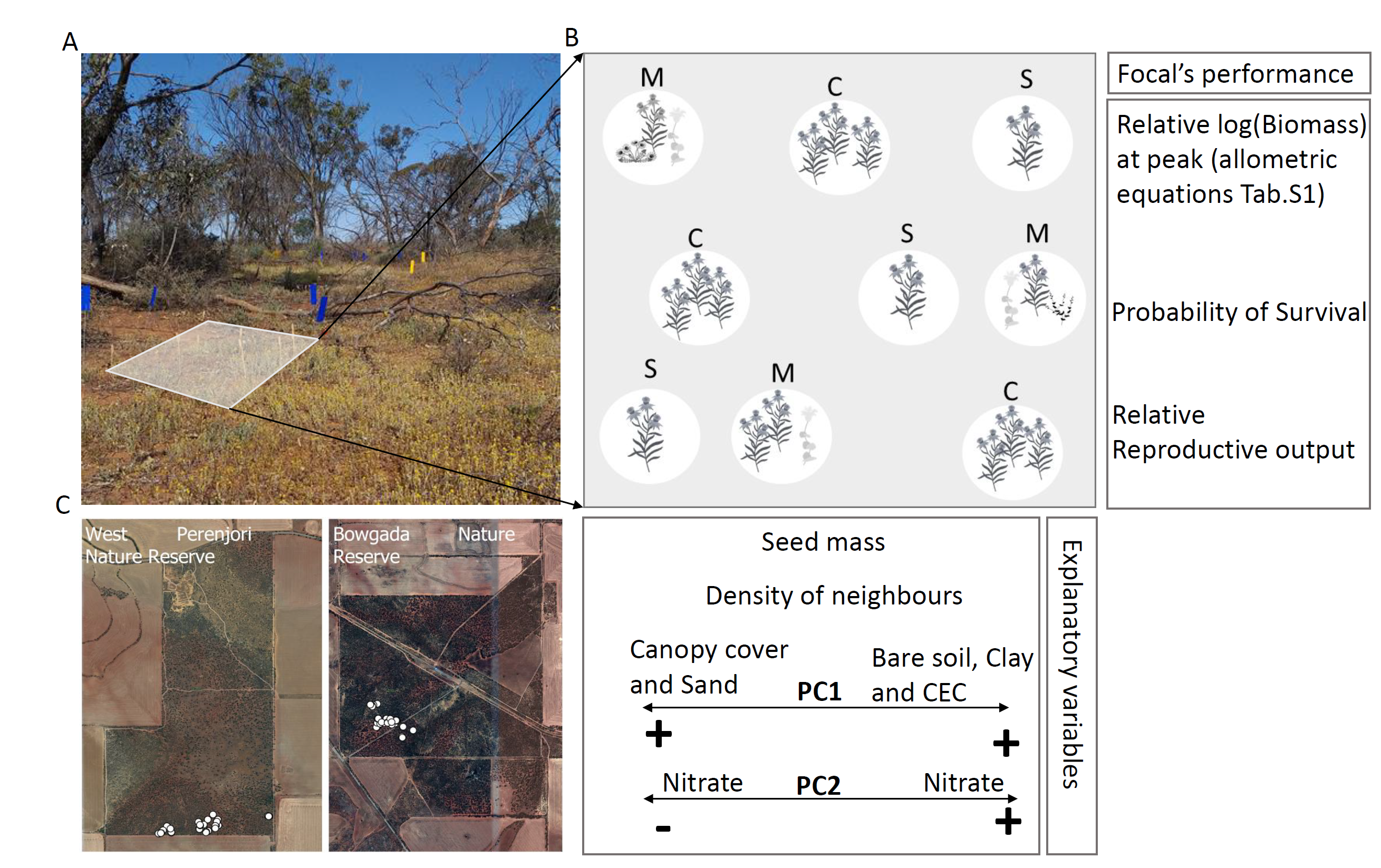


Fig. S1- Experimental design. A) Shows a plot located in West Perenjori Nature Reserve (photo by I. da Silva). B) Carton showing 15 cm of diameter circular plots within a plot (1 x 1 m), with 3 replicates of each treatment: solo plant (“S”); conspecifics only (“C”); heterospecifics and conspecifics (“M”=mixed neighbours). For each focal species, 12 plots were placed across a natural gradient of conspecifics densities (flower cartoons by Loy Xingwen). C) All experimental plots across West Perenjori Nature Reserve and Bowgada Nature Reserve, WA (Cartographic image from Google Earth, 2021).


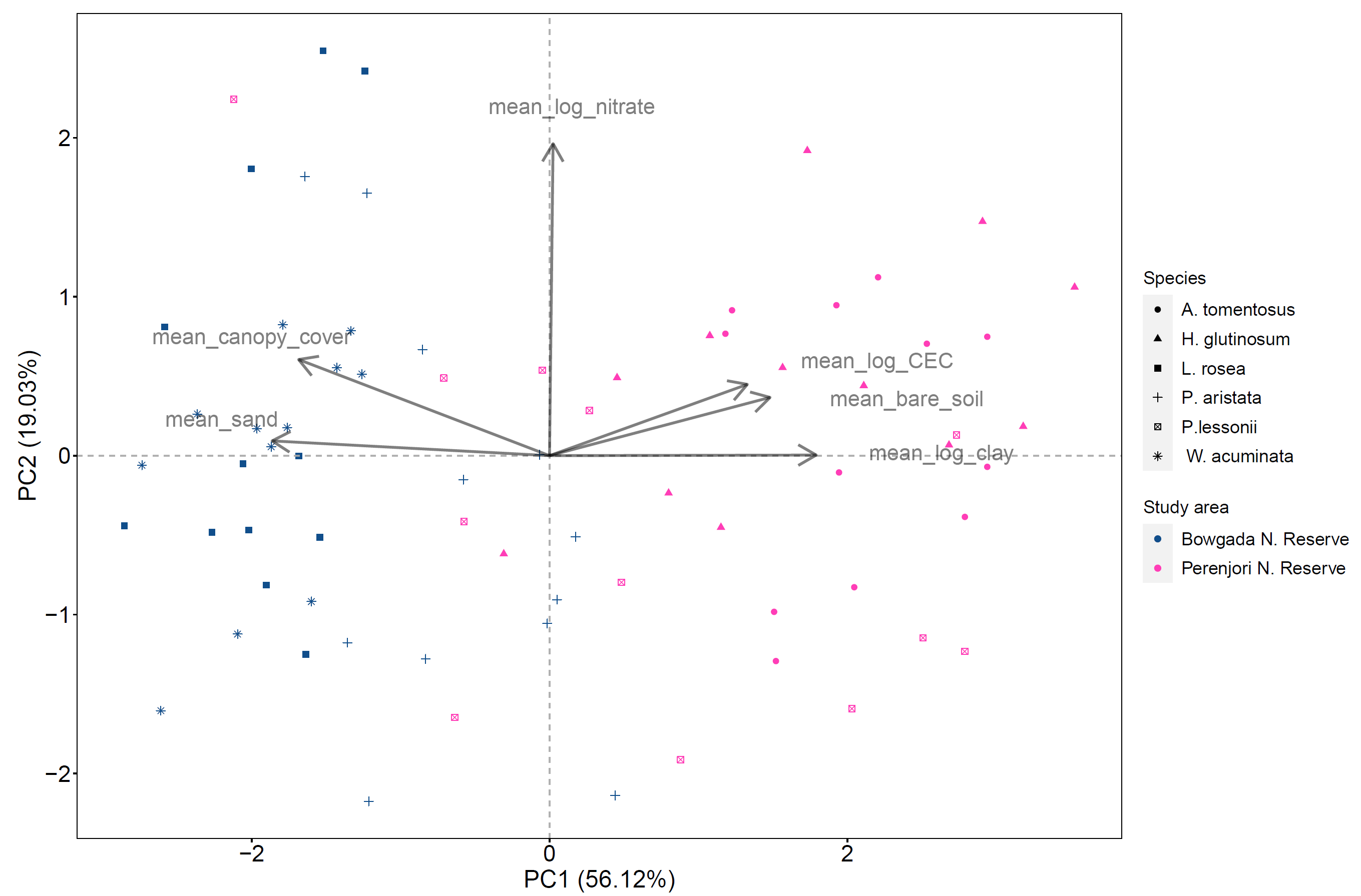


Fig. S2- Principal Component Analyses (PCA) showing the environmental gradient in relation to studied species and study localities. Environmental variables were: nitrate (mg/Kg), Cation exchange capacity (CEC), clay (%), sand (%), canopy cover (%) and bare soil (%).


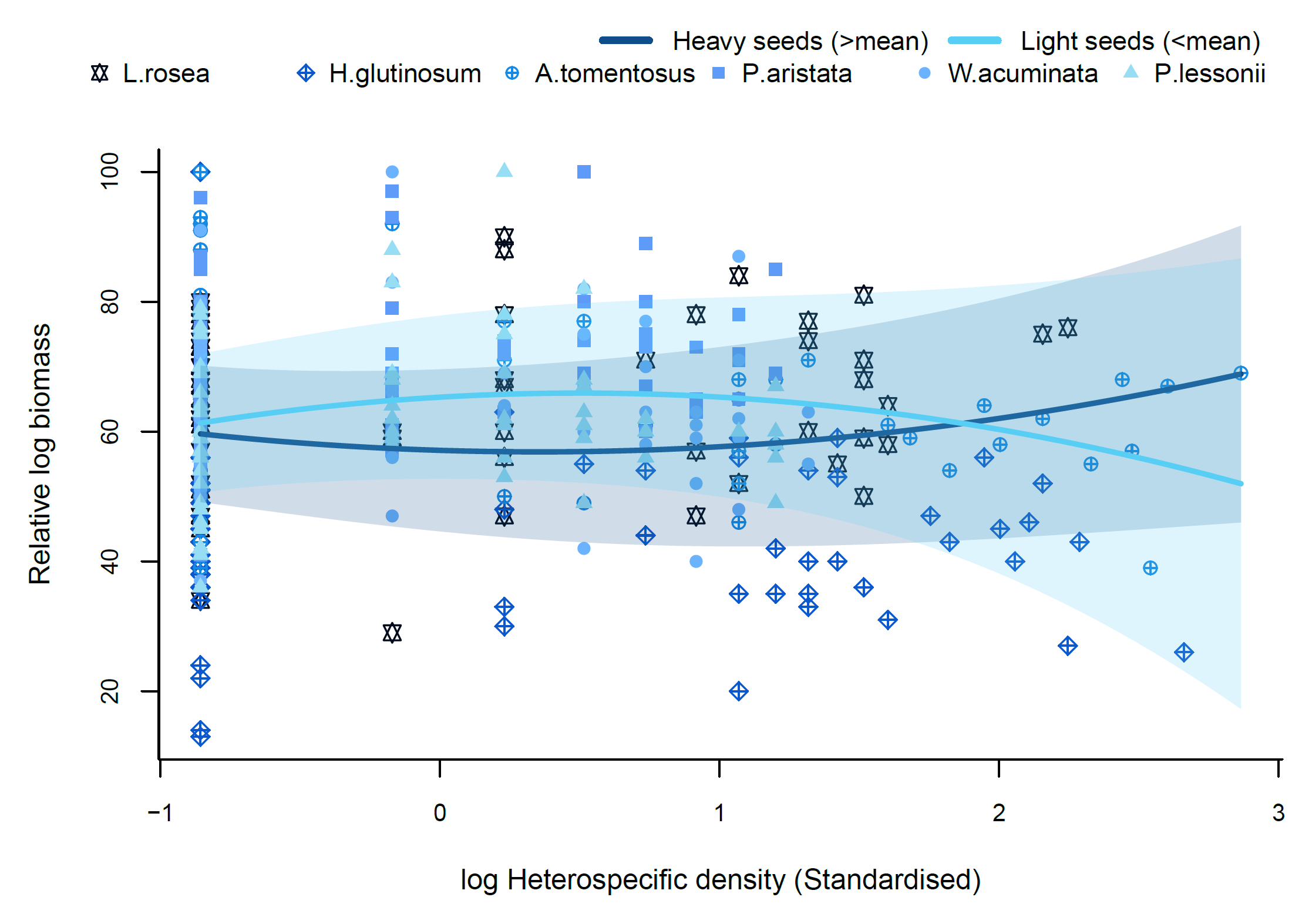


Fig. S3 Bivariate plots from final multilevel regression models for plots containing mixtures of conspecifics and heterospecifics (“M”) showing the relationship between relative log(biomass) and increasing densities of heterospecifics, with separate fitted lines for light-seeded and heavy-seeded species. Shaded areas in both plots represent ± 95% confidence intervals. Colours for species run from the darkest/heaviest (L. rosea = 1.278 mg) to the lightest (P. lessonii = 0.031 mg).

Table S1 Biomass (mg) allometric equations for 6 annual understory species from York gum-jam woodlands in Western Australia.

| Species | Allometric equation | Model  R-squared | Number of observations |
| --- | --- | --- | --- |
| *Angianthus tomentosus* | Biomass=1.090+log(height)*1.335+NF*0.052+NS*0.135 | 0.86 | 128 |
| *Hyalosperma glutinosum* | Biomass=-0.793+log(height)*1.036+NS*0.396 | 0.73 | 133 |
| *Lawrencella rosea* | Biomass=-0.119+log(height)*1.372+NF*0.346 | 0.74 | 130 |
| *Panaetia lessonii* | Biomass=2.327+log(height)*0.637+NF_NB*0.170 | 0.79 | 133 |
| *Podolepis aristata* | Biomass=2.542+log(height)*0.733+NF*0.225 | 0.76 | 133 |
| *Waitzia acuminata* | Biomass=1.832+log(height)*1.117+NF_NB*0.109 | 0.83 | 130 |
| NF= number of flowers; NS= number of stems; NB= number of buds, NL=number of leaves; NF_NB= number of flowers plus number of buds | | | |

Table S2 Principal Component Analyses (PCA) weights for the six environmental variables: nitrate (mg/Kg), Cation exchange capacity (CEC), clay (%), sand (%), canopy cover (%) and bare soil (%).

| Environmental Variables | PC1 | PC2 |
| --- | --- | --- |
| mean_log_nitrate | 0.012 | 0.982 |
| mean_log_CEC | 0.664 | 0.224 |
| mean_log_clay | 0.895 | 0.002 |
| mean_sand | -0.931 | 0.046 |
| mean_canopy_cover | -0.842 | 0.302 |
| mean_bare_soil | 0.740 | 0.183 |

Table S3 Within species confidence intervals and marginal means or probabilities estimates (emmeans package in R) for performance variables for three density treatments: solo plants (“S”), plants growing conspecifics (“C”) and plants growing with heterospecifics and conspecifics (“M”).

| *Response variables* | Rel. log(Biomass) | | | | | Probability of survival | | | | | Rel. reproductive output | | | | |
| --- | --- | --- | --- | --- | --- | --- | --- | --- | --- | --- | --- | --- | --- | --- | --- |
| *Species/ Treatment* | *emmean* | *SE* | *df* | *Lower CI* | *Upper CI* | *emmean* | *SE* | *df* | *Lower CI* | *Upper CI* | *emmean* | *SE* | *df* | *Lower CI* | *Upper CI* |
| *A. tomentosus* | | | | | | | | | | | | | | | |
| C | 63.813 | 2.493 | 66.679 | 58.837 | 68.789 | 0.902 | 0.052 | NA | 0.745 | 0.967 | 17.801 | 3.578 | 510.000 | 10.771 | 24.831 |
| M | 61.616 | 2.701 | 72.910 | 56.233 | 66.998 | 0.797 | 0.089 | NA | 0.573 | 0.920 | 14.479 | 4.135 | 510.000 | 6.355 | 22.603 |
| S | 69.465 | 2.990 | 69.459 | 63.502 | 75.429 | 0.813 | 0.081 | NA | 0.604 | 0.926 | 26.347 | 3.859 | 510.000 | 18.767 | 33.928 |
| *H. glutinosum* | | | | | | | | | | | | | | | |
| C | 39.124 | 2.486 | 67.295 | 34.162 | 44.087 | 0.707 | 0.087 | NA | 0.515 | 0.846 | 5.542 | 3.882 | 510.000 | -2.084 | 13.169 |
| M | 43.466 | 2.560 | 65.622 | 38.353 | 48.579 | 0.819 | 0.082 | NA | 0.606 | 0.930 | 6.227 | 4.043 | 510.000 | -1.716 | 14.170 |
| S | 40.643 | 2.960 | 67.863 | 34.736 | 46.550 | 0.664 | 0.106 | NA | 0.438 | 0.833 | 9.952 | 4.185 | 510.000 | 1.729 | 18.175 |
| *L. rosea* | | | | | | | | | | | | | | | |
| C | 67.124 | 2.487 | 67.296 | 62.161 | 72.086 | 0.682 | 0.090 | NA | 0.487 | 0.828 | 0.682 | 3.786 | 510.000 | 21.632 | 36.509 |
| M | 64.945 | 2.609 | 69.023 | 59.740 | 70.151 | 0.567 | 0.117 | NA | 0.340 | 0.769 | 0.567 | 4.523 | 510.000 | 14.657 | 32.430 |
| S | 61.866 | 2.953 | 63.242 | 55.967 | 67.766 | 0.847 | 0.075 | NA | 0.639 | 0.945 | 0.847 | 3.907 | 510.000 | 2.276 | 17.626 |
| *P. aristata* | | | | | | | | | | | | | | | |
| C | 69.563 | 2.448 | 63.700 | 64.673 | 74.454 | 0.927 | 0.044 | NA | 0.779 | 0.978 | 17.488 | 3.535 | 510.000 | 10.543 | 24.432 |
| M | 72.629 | 2.505 | 60.807 | 67.620 | 77.637 | 0.940 | 0.042 | NA | 0.787 | 0.985 | 20.512 | 3.836 | 510.000 | 12.976 | 28.048 |
| S | 67.007 | 2.905 | 63.317 | 61.202 | 72.812 | 0.938 | 0.042 | NA | 0.784 | 0.984 | 18.154 | 3.701 | 510.000 | 10.883 | 25.425 |
| *P. lessonii* | | | | | | | | | | | | | | | |
| C | 61.342 | 2.429 | 62.130 | 56.486 | 66.198 | 0.848 | 0.065 | NA | 0.676 | 0.937 | 0.848 | 3.638 | 510.000 | 5.480 | 19.774 |
| M | 64.739 | 2.505 | 60.807 | 59.730 | 69.748 | 0.895 | 0.060 | NA | 0.710 | 0.967 | 0.895 | 3.944 | 510.000 | 5.884 | 21.381 |
| S | 57.414 | 2.880 | 62.157 | 51.657 | 63.170 | 0.915 | 0.051 | NA | 0.747 | 0.975 | 0.915 | 3.701 | 510.000 | 2.325 | 16.868 |
| *W. acuminata* | | | | | | | | | | | | | | | |
| C | 64.304 | 2.467 | 65.427 | 59.378 | 69.230 | 0.930 | 0.043 | NA | 0.783 | 0.980 | 12.791 | 3.535 | 510.000 | 5.847 | 19.735 |
| M | 64.493 | 2.523 | 62.303 | 59.451 | 69.536 | 0.982 | 0.020 | NA | 0.858 | 0.998 | 16.915 | 3.807 | 510.000 | 9.436 | 24.395 |
| S | 65.802 | 2.897 | 63.488 | 60.014 | 71.590 | 0.915 | 0.051 | NA | 0.746 | 0.975 | 16.471 | 3.732 | 510.000 | 9.138 | 23.804 |

Table S4 Within species average variance in performance for three density treatments: solo plants (“S”), plants growing conspecifics (“C”) and plants growing with heterospecifics and conspecifics (“M”). Significant p-values ((P<0.05) are in bold mask.

| *Response variables* | Probability of survival | | | | | | | Rel. log(Biomass) | | | | | | Rel. reproductive output | | | | |
| --- | --- | --- | --- | --- | --- | --- | --- | --- | --- | --- | --- | --- | --- | --- | --- | --- | --- | --- |
| *Species /Contrasts* | *Odds.ratio* | *SE* | *df* | *null* | *z.ratio* | *p.value* | *Estimate* | | *SE* | *df* | *t.ratio* | *p.value* | *Estimate* | | *SE* | *df* | *t.ratio* | *P.value* |
| *A. tomentosus* | | | | | | | | | | | | | | | | | | |
| C / M | 2.361 | 1.726 | NA | 1 | 1.174 | 0.240 | 2.197 | | 2.605 | 71.619 | 0.843 | 0.402 | 3.322 | | 4.120 | 510 | 0.806 | 0.420 |
| C / S | 2.124 | 1.650 | NA | 1 | 0.970 | 0.332 | -5.652 | | 2.749 | 72.018 | -2.056 | **0.043** | -8.546 | | 4.017 | 510 | -2.128 | **0.034** |
| M / S | 0.900 | 0.660 | NA | 1 | -0.144 | 0.885 | -7.850 | | 2.855 | 77.090 | -2.749 | **0.007** | -11.868 | | 4.258 | 510 | -2.787 | **0.006** |
| *H. glutinosum* | | | | | | | | | | | | | | | | | | |
| C / M | 0.534 | 0.326 | NA | 1 | -1.027 | 0.305 | -4.342 | | 2.461 | 66.666 | -1.764 | 0.082 | -0.685 | | 4.290 | 510 | -0.160 | 0.873 |
| C / S | 1.224 | 0.746 | NA | 1 | 0.333 | 0.739 | -1.519 | | 2.703 | 69.281 | -0.562 | 0.576 | -4.410 | | 4.578 | 510 | -0.963 | 0.336 |
| M / S | 2.293 | 1.588 | NA | 1 | 1.199 | 0.230 | 2.823 | | 2.699 | 70.503 | 1.046 | 0.299 | -3.725 | | 4.457 | 510 | -0.836 | 0.404 |
| *L. rosea* | | | | | | | | | | | | | | | | | | |
| C / M | 1.633 | 0.889 | NA | 1 | 0.901 | 0.367 | 2.178 | | 2.516 | 71.050 | 0.866 | 0.390 | 5.527 | | 4.576 | 510 | 1.208 | 0.228 |
| C / S | 0.388 | 0.269 | NA | 1 | -1.367 | 0.172 | 5.257 | | 2.700 | 62.863 | 1.947 | 0.056 | 19.120 | | 4.176 | 510 | 4.579 | **0.000** |
| M / S | 0.237 | 0.171 | NA | 1 | -1.997 | **0.046** | 3.079 | | 2.700 | 62.479 | 1.140 | 0.258 | 13.592 | | 4.577 | 510 | 2.970 | **0.003** |
| *P. aristata* | | | | | | | | | | | | | | | | | | |
| C / M | 0.809 | 0.749 | NA | 1 | -0.229 | 0.819 | -3.065 | | 2.366 | 58.689 | -1.295 | 0.200 | -3.025 | | 3.792 | 510 | -0.798 | 0.425 |
| C / S | 0.844 | 0.812 | NA | 1 | -0.176 | 0.860 | 2.556 | | 2.609 | 61.030 | 0.980 | 0.331 | -0.666 | | 3.814 | 510 | -0.175 | 0.861 |
| M / S | 1.044 | 1.058 | NA | 1 | 0.042 | 0.966 | 5.621 | | 2.578 | 58.654 | 2.180 | **0.033** | 2.359 | | 3.805 | 510 | 0.620 | 0.536 |
| *P. lessonii* | | | | | | | | | | | | | | | | | | |
| C / M | 0.654 | 0.485 | NA | 1 | -0.572 | 0.567 | -3.397 | | 2.347 | 57.068 | -1.447 | 0.153 | -1.005 | | 4.007 | 510 | -0.251 | 0.802 |
| C / S | 0.515 | 0.418 | NA | 1 | -0.817 | 0.414 | 3.928 | | 2.562 | 58.256 | 1.533 | 0.131 | 3.030 | | 3.904 | 510 | 0.776 | 0.438 |
| M / S | 0.787 | 0.702 | NA | 1 | -0.269 | 0.788 | 7.326 | | 2.549 | 57.457 | 2.874 | **0.006** | 4.036 | | 3.918 | 510 | 1.030 | 0.304 |
| *W. acuminata* | | | | | | | | | | | | | | | | | | |
| C / M | 0.246 | 0.307 | NA | 1 | -1.125 | 0.261 | -0.189 | | 2.403 | 61.396 | -0.079 | 0.937 | -4.124 | | 3.760 | 510 | -1.097 | 0.273 |
| C / S | 1.219 | 1.123 | NA | 1 | 0.215 | 0.830 | -1.498 | | 2.612 | 61.604 | -0.573 | 0.568 | -3.680 | | 3.836 | 510 | -0.959 | 0.338 |
| M / S | 4.959 | 6.343 | NA | 1 | 1.252 | 0.211 | -1.309 | | 2.581 | 59.159 | -0.507 | 0.614 | 0.444 | | 3.799 | 510 | 0.117 | 0.907 |

Table S5 Single-species regression models for Probability of Survival in response to conspecifics. CI = confidence interval. Significant effects (P<0.05) are in bold mask.

| *Predictors* | *Estimate* | *CI (95%)* | *P-value* | *Random effects* | |
| --- | --- | --- | --- | --- | --- |
| *A. tomentosus* | | | | | |
|  |  |  |  |  | |
| Intercept | 1.93 | -0.37 – 4.22 | 0.1 | σ^2^ | 3.29 |
| log(Conspecifics density) | 0.11 | -0.28 – 0.51 | 0.566 | τ_00_ | 0.00 _plot_ |
| Environment PC1 | -0.43 | -2.34 – 1.48 | 0.658 | Marginal R^2^ / Conditional R^2^ | 0.02 / 02 |
| Environment PC2 | 0.13 | -0.70 – 0.97 | 0.752 | Observations/N | 72/12 |
| *H. glutinosum* | | | | | |
| Intercept | 0.13 | -0.95 – 1.20 | 0.817 | σ^2^ | 3.29 |
| log(Conspecifics density) | 0.04 | -0.21 – 0.29 | 0.768 | τ_00_ | 0.11 _plot_ |
| Environment PC1 | 0.64 | -0.38 – 1.66 | 0.22 | Marginal R^2^ / Conditional R^2^ | 0.04 / 0.07 |
| Environment PC2 | -0.17 | -1.06 – 0.73 | 0.718 | Observations/N | 72/12 |
| *L. rosea* | | | | | |
| Intercept | 0.35 | -4.26 – 4.95 | 0.882 | σ^2^ | 3.29 |
| log(Conspecifics density) | -0.40 | -0.92 – 0.12 | 0.131 | τ_00_ | 1.38 _plot_ |
| Environment PC1 | -1.26 | -5.55 – 3.03 | 0.564 | Marginal R^2^ / Conditional R^2^ | 0.10 / 0.37 |
| Environment PC2 | 0.50 | -0.40 – 1.39 | 0.276 | Observations/N | 72/12 |
| *P. aristata* | | | | | |
| Intercept | 1.81 | 0.31 – 3.30 | **0.018** | σ^2^ | 3.29 |
| log(Conspecifics density) | 0.31 | -0.72 – 1.34 | 0.557 | τ_00_ | 0.00 _plot_ |
| Environment PC1 | -2.07 | -5.11 – 0.98 | 0.183 | Marginal R^2^ / Conditional R^2^ | 0.17 / 0.17 |
| Environment PC2 | 0.11 | -0.91 – 1.13 | 0.83 | Observations/N | 72/12 |
| *P. lessonii* | | | | | |
| Intercept | 2.36 | 1.17 – 3.55 | **<0.001** | σ^2^ | 3.29 |
| log(Conspecifics density) | -0.08 | -0.97 – 0.80 | 0.857 | τ_00_ | 0.00 _plot_ |
| Environment PC1 | -0.40 | -1.36 – 0.56 | 0.418 | Marginal R^2^ / Conditional R^2^ | 0.14 / 0.14 |
| Environment PC2 | 0.44 | -0.41 – 1.29 | 0.312 | Observations/N | 72/12 |
| *W. acuminata* | | | | | |
| Intercept | 1.88 | -2.60 – 6.35 | 0.412 | σ^2^ | 3.29 |
| log(Conspecifics density) | 0.28 | -0.66 – 1.21 | 0.561 | τ_00_ | 0.05 _plot_ |
| Environment PC1 | -0.32 | -4.43 – 3.79 | 0.878 | Marginal R^2^ / Conditional R^2^ | 0.15 / 0.17 |
| Environment PC2 | -1.01 | -3.04 – 1.01 | 0.328 | Observations/N | 72/12 |

Table 6 Single-species regression models for Relative log biomass in response to conspecifics. CI = confidence interval. Significant effects (P<0.05) are in bold mask.

| *Predictors* | *Estimate* | *CI (95%)* | *P-value* | *Random Effects* | |
| --- | --- | --- | --- | --- | --- |
| *A. tomentosus* | | | | | |
|  |  |  |  |  | |
| Intercept | 76.17 | 53.58 – 98.77 | **<0.001** | σ^2^ | 113.12 |
| log(Conspecifics density) | -1.79 | -3.60 – 0.02 | 0.052 | τ_00_ | 95.55 _plot_ |
| Environment PC1 | -6.86 | -25.97 – 12.26 | 0.475 | Marginal R^2^ / Conditional R^2^ | 0.12 / 0.52 |
| Environment PC2 | 4.71 | -3.56 – 12.98 | 0.259 | Observations/N | 61/12 |
| *H. glutinosum* | | | | | |
| Intercept | 42.39 | 32.55 – 52.22 | **<0.001** | σ^2^ | 115.41 |
| log(Conspecifics density) | -0.42 | -1.82 – 0.98 | 0.551 | τ_00_ | 54.40 _plot_ |
| Environment PC1 | 0.37 | -8.99 – 9.73 | 0.937 | Marginal R^2^ / Conditional R^2^ | 0.05 / 0.35 |
| Environment PC2 | -4.46 | -12.96 – 4.04 | 0.298 | Observations/N | 62/12 |
| *L. rosea* | | | | | |
| Intercept | 83.27 | 56.95 – 109.58 | **<0.001** | σ^2^ | 105.79 |
| log(Conspecifics density) | 1.02 | -1.27 – 3.32 | 0.376 | τ_00_ | 68.51 _plot_ |
| Environment PC1 | 18.36 | -5.43 – 42.14 | 0.128 | Marginal R^2^ / Conditional R^2^ | 0.10 / 0.46 |
| Environment PC2 | -0.16 | -5.20 – 4.87 | 0.948 | Observations/N | 64/12 |
| *P. aristata* | | | | | |
| Intercept | 66.21 | 60.18 – 72.24 | **<0.001** | σ^2^ | 68.05 |
| log(Conspecifics density) | 1.65 | -0.91 – 4.20 | 0.202 | τ_00_ | 25.95 _plot_ |
| Environment PC1 | -3.32 | -14.27 – 7.64 | 0.547 | Marginal R^2^ / Conditional R^2^ | 0.04 / 0.31 |
| Environment PC2 | 0.76 | -2.64 – 4.16 | 0.656 | Observations/N | 67/12 |
| *P. lessonii* | | | | | |
| Intercept | 57.67 | 53.03 – 62.30 | **<0.001** | σ^2^ | 136.46 |
| log(Conspecifics density) | 2.81 | -1.05 – 6.68 | 0.151 | τ_00_ | 14.79 _plot_ |
| Environment PC1 | -0.64 | -5.83 – 4.54 | 0.805 | Marginal R^2^ / Conditional R^2^ | 0.03 / 0.12 |
| Environment PC2 | 0.02 | -3.98 – 4.01 | 0.994 | Observations/N | 69/12 |
| *W. acuminata* | | | | | |
| Intercept | 71.09 | 54.63 – 87.55 | **<0.001** | σ^2^ | 68.34 |
| log(Conspecifics density) | -0.72 | -3.07 – 1.63 | 0.541 | τ_00_ | 20.69 _plot_ |
| Environment PC1 | 5.44 | -10.26 – 21.13 | 0.491 | Marginal R^2^ / Conditional R^2^ | 0.05 / 0.27 |
| Environment PC2 | -3.34 | -8.89 – 2.21 | 0.234 | Observations/N | 66/12 |

Table 7 Single-species regression models for Relative reproductive output in response to conspecifics. CI = confidence interval. Significant effects (P<0.05) are in bold mask.

| *Predictors* | *Estimate* | *CI (95%)* | *P-value* | *Random effects* |  |
| --- | --- | --- | --- | --- | --- |
| *A. tomentosus* | | | | | |
| Intercept | 3.67 | 2.58 – 4.75 | **<0.001** | σ^2^ | 0.41 |
| log(Conspecifics density) | -0.11 | -0.23 – 0.02 | 0.092 | τ_00_ | 0.17 _plot_ |
| Environment PC1 | -0.56 | -1.46 – 0.34 | 0.226 | Marginal R^2^ / Conditional R^2^ | 0.18 / 0.42 |
| Environment PC2 | 0.32 | -0.07 – 0.70 | 0.111 | Observations/N | 61/12 |
| *H. glutinosum* | | | | | |
| Intercept | 1.69 | 0.65 – 2.73 | **0.001** | σ^2^ | 0.55 |
| log(Conspecifics density) | -0.17 | -0.34 – -0.01 | **0.039** | τ_00_ | 0.58 _plot_ |
| Environment PC1 | 0.47 | -0.52 – 1.46 | 0.353 | Marginal R^2^ / Conditional R^2^ | 0.16 / 0.59 |
| Environment PC2 | -0.56 | -1.44 – 0.31 | 0.208 | Observations/N | 48/12 |
| *L. rosea* | | | | | |
| Intercept | 3.47 | 1.71 – 5.23 | **<0.001** | σ^2^ | 0.98 |
| log(Conspecifics density) | 1.74 | 0.44 – 3.05 | **0.009** | τ_00_ | 0 |
| log(Conspecifics density)^2 | -0.53 | -1.01 – -0.04 | **0.033** | Marginal R^2^ / Conditional R^2^ | 0.35 / 35 |
| Environment PC1 | 1.21 | -0.37 – 2.79 | 0.133 | Observations/N | 56/12 |
| Environment PC2 | 0.13 | -0.22 – 0.48 | 0.467 |  |  |
| *P. aristata* | | | | | |
| Intercept | 2.85 | 2.38 – 3.33 | **<0.001** | σ^2^ | 0.47 |
| log(Conspecifics density) | -0.06 | -0.32 – 0.20 | 0.65 | τ_00_ | 0.07 _plot_ |
| Environment PC1 | -0.18 | -0.95 – 0.58 | 0.637 | Marginal R^2^ / Conditional R^2^ | 0.05 / 0.17 |
| Environment PC2 | 0.09 | -0.12 – 0.31 | 0.397 | Observations/N | 66/12 |
| *P. lessonii* | | | | | |
| Intercept | 2.09 | 1.43 – 2.74 | **<0.001** | σ^2^ | 1.01 |
| log(Conspecifics density) | 0.16 | -0.35 – 0.67 | 0.542 | τ_00_ | 0.44 _plot_ |
| Environment PC1 | -1.22 | -1.99 – -0.45 | **0.002** | Marginal R^2^ / Conditional R^2^ | 0.35 / 0.55 |
| Environment PC2 | -0.38 | -0.96 – 0.19 | 0.192 | Observations/N | 63/12 |
| *W. acuminata* | | | | | |
| Intercept | 57.67 | 53.03 – 62.30 | **<0.001** | σ^2^ | 136.46 |
| log(Conspecifics density) | 2.81 | -1.05 – 6.68 | 0.151 | τ_00_ | 14.79 _plot_ |
| Environment PC1 | -0.64 | -5.83 – 4.54 | 0.805 | Marginal R^2^ / Conditional R^2^ | 0.09 / 0.14 |
| Environment PC2 | 0.02 | -3.98 – 4.01 | 0.994 | Observations/N | 69/12 |
